# Carbon dots derived from traditional Chinese medicines: a novel therapeutic strategy for ocular diseases

**DOI:** 10.3389/fphar.2026.1912653

**Published:** 2026-10-14

**Authors:** Tingyu Yang, Zongqi Feng, Xiao Zhang, Lihui Zhang, Yu Su, Ruihua Liu, Rina Sa, Lamusi A

**Affiliations:** 1 Scientific Research Department, Inner Mongolia Fourth Hospital (Chest Hospital), Hohhot, Inner Mongolia, China; 2 Department of Clinical Research and Translational Medicine, Inner Mongolia People’s Hospital, Hohhot, Inner Mongolia, China; 3 Department of Ophthalmology, Inner Mongolia International Hospital of Mongolian Medicine, Hohhot, Inner Mongolia, China

**Keywords:** academic controversies, carbon dots, ocular disease treatment, TCM-CDs, therapeutic mechanisms

## Abstract

Carbon dots (CDs) represent a novel category of zero-dimensional carbon-based nanomaterials featuring favorable biosafety *in vitro* and in acute animal studies. They also exhibit distinctive optical properties and diverse pharmacological activities. Synthesized via eco-friendly green routes using traditional Chinese medicines (TCMs) and their extracts, TCM-derived carbon dots (TCM-CDs) display distinct properties compared to conventional nanocarriers. Benefiting from their inherent nanostructures, abundant surface functional groups, and nanozyme-like activity, TCM-CDs can deliver intrinsic therapeutic effects without loading exogenous drugs or relying on residual herbal ingredients. They possess multiple bioactivities, including antioxidant, anti-inflammatory, antibacterial and neuroprotective effects, which make them potential non-invasive therapeutic candidates for refractory ocular disorders. A growing body of studies has validated the promising therapeutic efficacy of TCM-CDs in animal models against autoimmune uveitis, retinal lesions, and optic nerve injuries, highlighting their encouraging preclinical findings that warrant further investigation toward clinical translation. Focusing on the intrinsic therapeutic capacity of TCM-CDs, this review systematically summarizes their core functional mechanisms and research progress in ocular disease intervention, discusses prevailing academic controversies and unresolved research bottlenecks in this field, and proposes prospective developmental directions. This work intends to offer comprehensive theoretical evidence and guidance directions for subsequent mechanistic investigations and clinical translation of TCM-CDs in ophthalmic research.

## 1 Introduction

Ocular physiological barriers, including the corneal barrier and blood-retinal barrier, together with rapid tear clearance, drastically limit intraocular drug delivery efficiency (Nafar et al., 2026). Conventional eye drops exhibit extremely low ocular bioavailability and merely relieve clinical symptoms (Nafar et al., 2026). In contrast, systemic administration delivers unsatisfactory tissue targeting and triggers severe adverse reactions (Nafar et al., 2026). Long-term glucocorticoid exposure induces diverse adverse ocular reactions, whereas extended antibiotic therapy readily drives the emergence of drug resistance and secondary complications (Nafar et al., 2026). Traditional Chinese medicines (TCMs) possess unique merits in improving visual function, mitigating inflammation and protecting neural tissues (Polat et al., 2025). Nevertheless, conventional TCM preparations are restricted by poor water solubility, high molecular weight and insufficient ocular penetrability, all of which impede precise intraocular therapy. The emergence of nanotechnology has provided a feasible strategy to overcome these critical bottlenecks. TCM-derived carbon dots (TCM-CDs), fabricated from TCM raw materials and extracts via green carbonization approaches, are characterized by tiny particle size, abundant surface functional groups and nanozyme-like activity. They exhibit great application potential in scavenging reactive oxygen species (ROS), inhibiting inflammatory responses and preserving neural function (Wang et al., 2025; Sun et al., 2025; Chu et al., 2026).

To date, the intrinsic therapeutic activities of CDs and their pharmacological antioxidant and anti-inflammatory effects have been demonstrated in research fields such as gastrointestinal diseases, and systematic reviews have summarized the common mechanisms by which they regulate oxidative stress and inflammatory pathways (Beiranvand and Dehghan, 2025). Building on these findings, research on the intrinsic therapeutic activities of CDs in the treatment of ocular diseases has also been extensively conducted, with several relevant reviews having been published (Unnikrishnan et al., 2025; Zhang et al., 2024; Kumar et al., 2023). However, investigations focusing on ophthalmic applications of TCM-CDs remain in the preliminary exploratory stage. Current relevant reports are mostly discrete basic experiments covering only a narrow spectrum of ocular disorders, including diabetic retinopathy (DR) and autoimmune uveitis (AU), accompanied by prominent heterogeneity across different research works. To the best of our knowledge, no systematic review has been specifically dedicated to this direction to date.

Given the above research status, this review focuses on the intrinsic therapeutic activities of TCM-CDs in ocular diseases, systematically summarizing existing findings, generalizing core therapeutic mechanisms, and presenting progress in applications across different ocular conditions. Furthermore, this paper sorts out major academic disputes and technical bottlenecks regarding therapeutic activity origins, structure-activity relationships and ocular interactions, and delivers forward-looking insights for subsequent research. This review is expected to provide theoretical foundations and research guidance for follow-up ophthalmic studies of TCM-CDs, as well as innovative ideas and research avenues for developing TCM-CD-based ocular therapeutic agents.

To ensure conceptual clarity throughout this review, we propose the following operational definition: TCM-CDs refer to carbon nanomaterials synthesized using traditional Chinese medicinal materials-including crude herbs, herbal extracts, or purified bioactive constituents derived from TCM as carbon precursors via pyrolytic or hydrothermal carbonization approaches. Based on the nature of the precursor, TCM-CDs can be classified into two subcategories: Type I: Prepared from whole medicinal herbs. Type II: Prepared from herbal extracts or purified bioactive constituents derived from TCM.

## 2 Core mechanisms underlying the inherent therapeutic activities of TCM-CDs

The ocular therapeutic efficacy of TCM-CDs mainly stems from their nanoscale size effect, rich surface functional groups and inherent nanozyme-like catalytic activity. Multiple studies have verified that purified TCM-CDs (with residual small-molecule herbal impurities eliminated) still maintain stable pharmacological functions, which confirms that their nanostructures act as the core functional unit mediating therapeutic effects (Wang et al., 2025; Sun et al., 2025; Chu et al., 2026). Their core therapeutic mechanisms fall into four synergistic functional modules: antioxidant, anti-inflammatory, antibacterial and neuroprotective effects. Antioxidant function serves as the fundamental pathway to mitigate oxidative stress within pathological microenvironments; anti-inflammatory activity acts as the central hub to suppress inflammatory cascade amplification; antibacterial and neuroprotective effects further expand their application scope by targeting infectious and degenerative ocular lesions, respectively.

### 2.1 Antioxidant activity

Hydroxyl (-OH) and carboxyl (-COOH) groups on the CDs’ surface directly scavenge excess ROS and reactive nitrogen species (RNS) in the pathological microenvironment, while also exhibiting nanozyme-like activities such as superoxide dismutase (SOD) and catalase (CAT), effectively regulating oxidative stress balance (Xu et al., 2026; Gao et al., 2023; Liu et al., 2026). Wang et al. (2025) synthesized Scutellaria barbata and Herba hedyotis diffusae-derived CDs (SH-CDs, Type I) via a one-step hydrothermal method using Scutellaria barbata and Herba hedyotis as precursors. The resulting SH-CDs have an average size of ∼2.1 nm, are rich in oxygen-containing groups (-OH, -COOH), and exhibit excellent ROS/RNS scavenging ability and SOD-like nanozyme activity. Topical eye-drop delivery of SH-CDs achieves corneal penetration in animal models, allowing SH-CDs to accumulate in the lens and retina. They further clear excessive high glucose-induced ROS and remarkably slow the progression of DR and diabetic cataract (DC) (Figure 1A).

**FIGURE 1:**
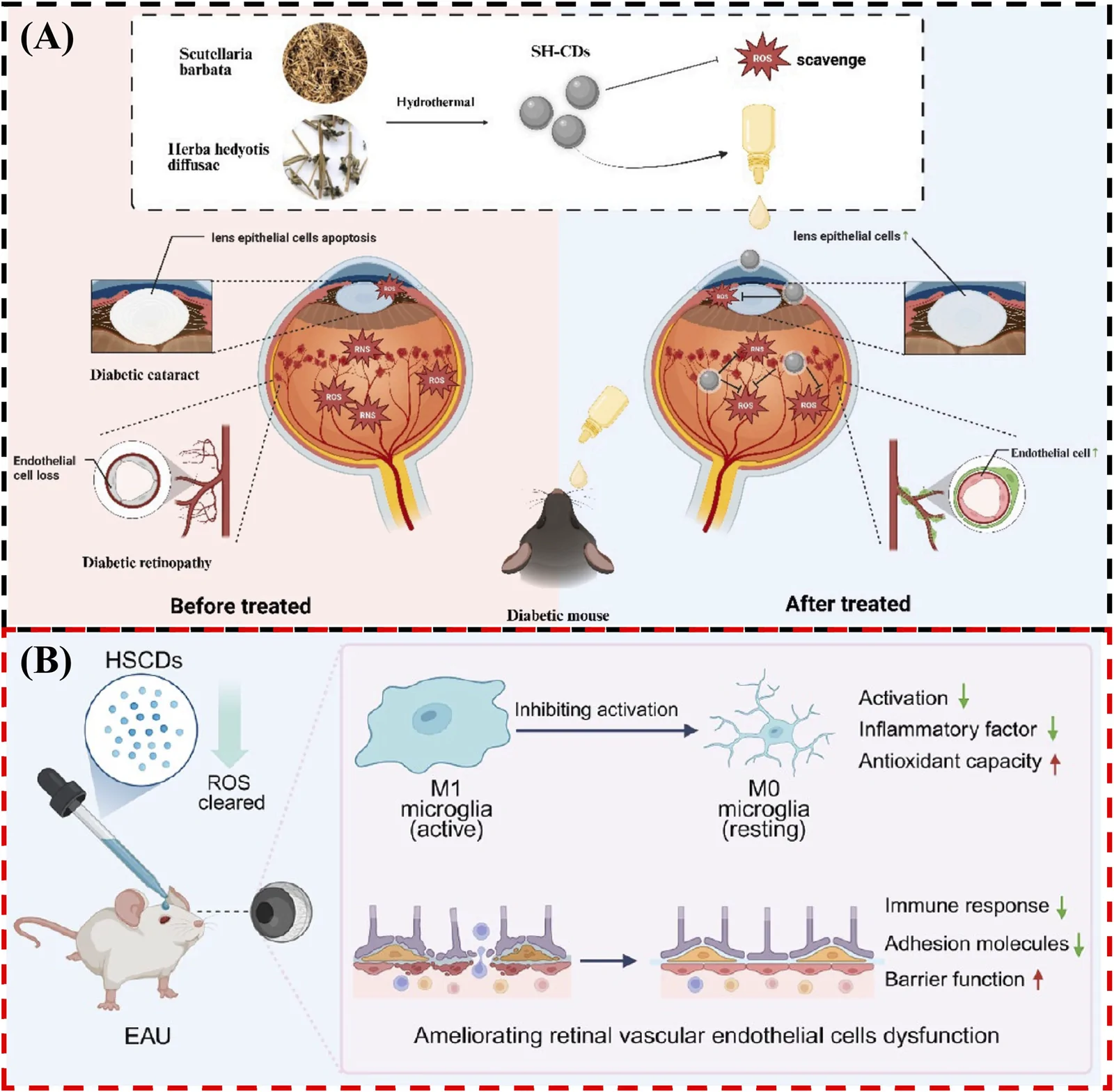
**(A)** Synthesis of SH-CDs and unified mechanism against DC and DR progression. Reprinted with permission from Ref (Wang et al., 2025). Copyright 2025, Elsevier. **(B)** Mechanism underlying non-invasive topical ocular administration of HSCDs inhibiting EAU progression. Reprinted with permission from Ref (Chu et al., 2026). Copyright 2026, American Chemical Society.

Additionally, Sun et al. (Sun et al., 2025) prepared curcumin-derived carbon dots (Cur-CDs, Type II) via a mild pyrolysis method using the TCM active ingredient curcumin (Cur) as a precursor. Surface moieties identified included cinnamic acid-, anisole-, guaiacol-, and ferulic acid-like structures. Notably, Cur-CDs effectively suppress H_2_O_2_-induced intracellular ROS production *in vitro*. In a rat model of retinal ischemia-reperfusion injury, Cur-CDs significantly reduce apoptosis, inflammation, and endoplasmic reticulum stress, with superior effects compared with equivalent concentrations of free Cur. Mechanistic investigations demonstrate that Cur-CDs exert protective effects by upregulating BCL-2 and HO-1 while downregulating CHOP and caspase-3. This evidence directly validates that the nanostructure of TCM-CDs constitutes the core functional unit responsible for antioxidant capacity. This antioxidant capacity not only directly protects ocular tissues but also serves as the upstream initiator for subsequent anti-inflammatory and neuroprotective effects, establishing a hierarchical cascade of therapeutic actions.

### 2.2 Anti-inflammatory activity

TCM-CDs can target and modulate canonical inflammatory signaling cascades (e.g., Nrf2/HO-1 and NF-κB), suppress abnormal microglial activation, and decrease the secretion of pro-inflammatory cytokines such as TNF-α, IL-6 and IL-1β (Chu et al., 2026). Chu et al. (Chu et al., 2026) prepared Scutellaria barbata and Hedyotis diffusae herba-derived CDs (HSCDs, Type I) using Scutellaria barbata and Hedyotis diffusae herba as synergistic precursors. In mouse models of experimental autoimmune uveitis (EAU), topical HSCDs eye drops exert dual therapeutic functions. On one hand, they block M1-type microglial polarization by activating the Nrf2/HO-1 axis. On the other hand, the SOD-mimicking nanozyme activity of HSCDs directly eliminates excess ROS, alleviating inflammatory reactivity of retinal vascular endothelial cells and restoring retinal vascular homeostasis (Figure 1B). Dual regulatory effects of HSCDs yield prominent therapeutic improvements in EAU mice without obvious biosafety risks. Furthermore, Deng et al. (Deng et al., 2025) fabricated honeysuckle-derived TCM-CDs (Type I), which also exhibit SOD-like catalytic activity, efficiently scavenging ROS and exerting anti-inflammatory effects in a lung inflammation model by inhibiting the Caspase-11/GSDMD-dependent pyroptosis pathway. This pharmacological mechanism offers critical references for ocular anti-inflammatory applications of TCM-CDs. Building upon oxidative stress alleviation, the anti-inflammatory action serves as the central hub that halts the progression of inflammatory cascades.

### 2.3 Broad-spectrum antibacterial activity

Positively charged CDs electrostatically bind to the negatively charged membranes of bacteria, disrupting membrane structural integrity and triggering intracellular ROS explosion. This mechanism enables potent elimination of multidrug-resistant ocular pathogens while avoiding the emergence of drug resistance (Vadakkan et al., 2025; Jian et al., 2017). Jian et al. (Jian et al., 2017) synthesized super-cationic CDs via a one-step dry heating strategy using spermidine as a precursor. These CDs carry abundant positive charges and exhibit strong bactericidal activity against both multidrug-resistant and drug-susceptible bacteria. The underlying mechanism relies on electrostatic adsorption-mediated destruction of bacterial cell membranes, which ultimately causes complete membrane collapse. Topical administration of these CDs transiently opens corneal epithelial tight junctions to boost corneal penetrability, and achieves remarkable therapeutic efficacy in rabbit models of bacterial keratitis. Although spermidine itself is not a TCM ingredient, it naturally exists in Lycium barbarum and other medicinal herbs (Ahad et al., 2020). This finding provides a feasible strategy for constructing novel antibacterial TCM-CDs from herbal extracts.

### 2.4 Neuroprotective and anti-apoptotic activity

TCM-CDs alleviate optic nerve injury caused by ischemia and inflammation by blocking apoptotic pathways of retinal ganglion cells, regulating the polarization balance of microglia and scavenging excessive ROS. After topical delivery, SH-CDs penetrate the cornea and accumulate within retinal tissues, where they clear hyperglycemia-induced ROS, suppress oxidative stress-triggered cellular apoptosis, and preserve normal retinal neuronal function (Wang et al., 2025). Cur-CDs deliver synergistic neuroprotective effects by modulating oxidative stress, endoplasmic reticulum stress and apoptotic signaling by upregulating BCL-2/HO-1 and downregulating CHOP/caspase-3 expression (Sun et al., 2025).

## 3 Research advances of TCM-CDs in ocular disease treatment

With the rapid progress of fundamental investigations, TCM-CDs have been adopted to intervene in various ocular disorders, and their therapeutic potential has been validated in multiple animal disease models. To facilitate comparative analysis of research designs, functional pathways and translational prospects across existing studies, representative research works concerning TCM-CDs are categorized and summarized in Table 1.

**TABLE 1:** Summary of representative TCM-CDs for ocular disease treatment.

TCM-CDs	Carbon source	Type	Method	Size (nm)	Zeta potential (mV)	Morphology	Optical properties	Purification method	Ocular disease model	Administration regimen	Treatment duration	Primary mechanism	Safety findings	References
SH-CDs	Scutellaria barbata and herba hedyotis diffusae	I	Hydrothermal (180 °C, 18 h)	∼2.1	NR	Spherical, d = 0.21 nm	FL (Ex 347/Em 426 nm), QY 7.9%; UV-Vis: 275 nm	Dialysis (MWCO 1 kDa) + 0.22 µm filtration	DR and DC	Topical eye drops,1 mg/mL, 4×/day	12 weeks	Antioxidant (SOD-like nanozyme activity, scavenges ROS/RNS), anti-inflammatory, anti-apoptotic	No ocular/systemic toxicity; normal blood counts and liver/kidney function	Wang et al. (2025)
Cur-CDs	Curcumin	II	Pyrolysis (150 °C, 2 h)	∼4.5	> −30	Crystalline sp2 carbon, d = 0.21 nm	FL (Ex 365 nm, 400–600 nm), QY < 1%; UV-Vis: 275 nm	Dialysis + alkaline treatment	Retinal I/R injury	Intravitreal injection,100 μg/mL	3 days post-injection before I/R induction, sampling at 24 h post-I/R; ERG up to 28 days	Antioxidant (scavenges ROS, upregulates HO-1), anti-inflammatory, anti-apoptotic (upregulates BCL-2, downregulates CHOP/caspase-3)	No retinal toxicity (ERG at 7/28 days); retinal structure preserved (H&E); >80% viability at 100 μg/mL	Sun et al. (2025)
HSCDs	Scutellaria barbata and hedyotis diffusae herba	I	Hydrothermal (180 °C, 12 h)	∼2.21	NR	Monodisperse spherical	UV-Vis: 277 nm; FL (2D/3D)	Dialysis (MWCO 1 kDa) + 0.22 µm filtration	EAU	Topical eye drops, 100 μg/mL, 4×/day	7 days (days 7–14)	Antioxidant (SOD-like nanozyme activity, scavenges ROS), anti-inflammatory (activates Nrf2/HO-1, inhibits microglial M1 polarization)	No ocular/systemic toxicity (14/30 days); low immunogenicity; endotoxin/β-glucan negative	Chu et al. (2026)

Abbreviations: NR, not reported; FL, fluorescence; QY, quantum yield; I/R, ischemia-reperfusion; EAU, experimental autoimmune uveitis.

### 3.1 Ocular surface diseases

Ocular surface disorders mainly consist of dry eye disease (DED) and infectious keratitis; the core pathological features of DED are disrupted oxidative homeostasis and chronic low-grade inflammation on the ocular surface (Krawczyk et al., 2024). No direct research has yet applied TCM-CDs to treat DED. However, Yang et al. (2025) prepared a thermoresponsive antioxidant carbon dot hydrogel (F-CD hydrogel) from citric acid and hydrazine, which exerted therapeutic effects in a mouse DED model by scavenging multiple free radicals, stabilizing mitochondrial membrane potential, and promoting corneal epithelial repair. This work offers valuable technical references for developing TCM-CD-based therapies against DED.

Infectious keratitis is predominantly induced by bacterial pathogens (Ather and Conrady, 2026), yet there are still no direct reports regarding TCM-CDs for this disorder. Notably, Wu et al. (Wu et al., 2026) used Cur (a TCM extract) and chitosan as precursors to prepare CDs via a hydrothermal method and constructed a photo-responsive composite hydrogel (CHB@CC). Compared with free Cur, the water solubility of the CDs is enhanced >4000-fold; they possess both SOD- and CAT-like activities, killing bacteria via electrostatic membrane disruption while scavenging ROS to protect host cells. In a rabbit bacterial keratitis model, this composite system reduces bacterial load by 90% within 24 h, maintains >98% cell viability, promotes M2-type anti-inflammatory macrophage polarization, suppresses fibrosis markers by 91.8%, and restores 87.5% of retinal signal amplitude, achieving synergistic antibacterial, anti-inflammatory, and corneal repair effects. This landmark study delivers critical references for the formulation optimization and multifunctional synergistic design of TCM-CDs. Collectively, research on TCM-CDs for ocular surface disorders remains in the preliminary stage and demands more in-depth investigations.

### 3.2 Autoimmune uveitis

Autoimmune uveitis (AU) is an immune-mediated intraocular inflammatory disorder and a leading contributor to visual loss. Clinically, long-term dependence on hormonal treatment inevitably triggers severe adverse reactions (Li et al., 2022). Topical eye drop delivery of HSCDs alleviates EAU by virtue of their inherent antioxidant and anti-inflammatory properties, yielding prominent therapeutic outcomes with favorable biosafety profiles (Chu et al., 2026).

### 3.3 Retinal and optic nerve diseases of the fundus

DR is pathologically featured by accumulated retinal oxidative stress, disrupted vascular barriers and abnormal neovascularization (Che et al., 2026; Mimura and Noma, 2025). Topically administered SH-CDs eliminate high glucose-induced redundant ROS and slow the pathological progression of DR and DC (Wang et al., 2025). Furthermore, Cur-CDs markedly inhibit retinal ganglion cell apoptosis in retinal ischemia-reperfusion injury models (Sun et al., 2025). Since retinal ischemia-reperfusion injury mimics the pathological processes of ischemic retinopathies such as glaucoma and retinal artery occlusion (Coviltir et al., 2024), Cur-CDs hold great potential for neuroprotective therapy of related diseases.

## 4 Core academic controversies regarding the therapeutic effects of TCM-CDs

The therapeutic potential of TCM-CDs against ocular disorders has been preliminarily verified, yet their detailed molecular mechanisms are still far from fully clarified. Current academic disputes mainly center on three core dimensions: the origin of therapeutic activity, structure-activity relationships, and ocular interactions.

### 4.1 Activity sources

This debate revolves around whether therapeutic efficacy originates from the intrinsic nanostructure of TCM-CDs or residual small-molecule herbal impurities. In routine preparation, TCM-CDs undergo dialysis and other steps to remove unreacted small molecules. Purified HSCDs alone exhibit significant anti-inflammatory activity (Chu et al., 2026), and Cur-CDs show superior pharmacological activity compared to equivalent concentrations of free Cur (Sun et al., 2025). This evidence confirms that the nanoscale effects, surface functional groups, and nanozyme-like activities of TCM-CDs are the core basis for their therapeutic effects, rather than reliance on residual herbal small molecules. However, given the high specific surface area and abundant surface functional groups of TCM-CDs, it cannot be excluded that small TCM molecules adsorbed on the surface or encapsulated inside TCM-CDs also participate in therapeutic effects and act synergistically with the nanostructures of TCM-CDs. In other words, the overall efficacy may result from the combined action dominated by nanostructures and assisted by small drug molecules, which requires further systematic research.

For future investigations in this field, we recommend researchers adopt rigorous purification workflows and perform quantitative detection of residual herbal precursors. Appropriate control groups, including purified TCM-CDs, free herbal precursors, and equivalent-concentration physical mixtures, should be included to better distinguish the biological contributions originating from carbon-dot nanostructures versus residual small-molecule components.

### 4.2 Structure-activity relationships

The core of this controversy is to clarify which structural characteristics determine the efficacy of TCM-CDs. Existing studies demonstrate that surface oxygen-containing groups (-OH and -COOH) serve as catalytic sites for SOD activity and are closely associated with antioxidant capacity (Wang et al., 2025; Chu et al., 2026), whereas surface positive charge density is positively correlated with antibacterial activity (Jian et al., 2017). Nevertheless, systematic quantitative structure-activity relationship (QSAR) models have not yet been established, and the generalizability of structural functional rules across TCM-CDs from different herbal precursors remains elusive. These limitations hinder the targeted structural optimization of TCM-CDs and the exploration of correlations between their structures and therapeutic efficacy.

### 4.3 Ocular interactions

This controversy aims to clarify how TCM-CDs interact with ocular cells at both the physical and molecular levels. Regarding physical delivery, particle size alone cannot guarantee biologically effective tissue penetration. It is critical to distinguish three separate concepts: corneal penetration, intraocular tissue distribution, and complete crossing of the blood-retinal barrier. Current tissue enrichment results cannot serve as direct evidence for full translocation across the intact blood-retinal barrier. In addition, most existing studies cannot readily distinguish whether the detected signal originates from intact TCM-CDs, degradation fragments, or dissociated small-molecule products. Beyond these physical barriers to delivery, the molecular targets of TCM-CDs also remain to be elucidated. At the signaling pathway level, TCM-CDs can regulate key pathways including Nrf2/HO-1 (Chu et al., 2026) and BCL-2/caspase-3 (Sun et al., 2025); at the cellular level, they inhibit M1 polarization of microglia and protect retinal vascular barriers (Chu et al., 2026). It should be noted that most current evidence describing TCM-CD-mediated modulation of the above-mentioned signaling pathways is based on observational changes in protein or gene expression levels, which represent correlative findings rather than definitive causal proof. Few studies have performed specific pathway inhibition, gene silencing or overexpression experiments to validate causal relationships. Further intervention-based mechanistic investigations are required to discriminate correlative biomarker alterations from true molecular regulatory effects. Resolving these interconnected academic disputes will lay a solid theoretical foundation for the clinical translation of TCM-CDs.

## 5 Research bottlenecks and future perspectives

### 5.1 Existing research bottlenecks

Beyond the above academic disputes, multiple critical research bottlenecks persist in current research systems. First, the *in vivo* research system for ophthalmic TCM-CD applications is incomplete. Most current investigations only conduct *in vitro* functional assessment and short-term toxicological tests using small animal models. Comprehensive data regarding intraocular distribution, metabolic clearance and tissue accumulation of TCM-CDs are scarce, and long-term chronic toxicology research is almost absent. Moreover, systematic evaluations focusing on dose-dependent toxicity, ocular irritation responses and systemic exposure risks are still insufficient. Notably, pre-clinical observations obtained from small-animal studies cannot be directly extrapolated to human conditions; rigorous long-term safety evaluation therefore constitutes an indispensable prerequisite before any clinical translation of TCM-CDs. Second, unified standards for preparation and evaluation have not been established. Research groups adopt different carbon sources, synthesis protocols, purification methods and characterization techniques, leading to poor repeatability and comparability of experimental results and restricting the systematic development of this field. Third, clinical transformation lags far behind. All current studies remain at the basic research stage, and no clinical trials on TCM-CDs for ocular disease treatment have been launched.

### 5.2 Future development directions

To address the above academic controversies and research bottlenecks, future research can focus on the following directions. In terms of mechanistic research, standardized purification protocols should be established. Meanwhile, combined with multi-omics analytical technologies, researchers can further decode the molecular interaction mechanisms between TCM-CDs, ocular cells and pathological signaling cascades. In particular, systematic QSAR models should be constructed to clarify the quantitative correlations between particle size, surface functional groups and therapeutic activities, so as to realize targeted structural optimization of TCM-CDs.

In terms of technical development, surface functional modification can be applied to improve the targeting ability of TCM-CDs. Recent studies have successfully constructed a hyaluronic acid-targeted nanosystem for breast cancer theranostics by integrating CDs with Fe_3_O_4_ nanoparticles and achieving active targeting through surface modification, demonstrating the feasibility of this strategy (Beiranvand et al., 2025). Inspired by this approach, conjugating appropriate targeting ligands to the surface of TCM-CDs is expected to enhance their targeting capability toward specific ocular tissues and cells. In addition, pathological responsive intelligent TCM-CDs or multifunctional ocular therapeutic preparations combined with other biomaterials can be developed. Moreover, large animal experiments on long-term safety and pharmacokinetics should be performed, and specialized preparation and quality control standards for ophthalmic TCM-CDs need to be formulated.

Apart from structural engineering of TCM-CDs, expanding the diversity of carbon precursor materials represents another promising research direction. Beyond conventional TCMs, the preparation technology of TCM-CDs can be extended to ethnic minority medicines including Mongolian medicine and Tibetan medicine. These ethnic medicines have complete theoretical systems, and many medicinal materials (such as saffron, sea buckthorn and chebula) possess efficacies of clearing heat, improving eyesight, activating blood circulation and detoxifying, showing unique advantages in the treatment of chronic and refractory ocular diseases (Mohtashami et al., 2021; Nakamura et al., 2017; Suryavanshi et al., 2022). Beyond single herbal precursors, fabricating TCM-CDs using compound prescriptions of traditional Chinese and ethnic minority medicines as mixed carbon sources deserves in-depth exploration. Compound prescription-derived TCM-CDs are expected to inherit the multi-target and holistic regulatory properties of traditional herbal formulas, while simultaneously acquiring strengthened ocular penetrability and nanozyme catalytic activity. Breakthroughs in this research direction will drive the evolution of TCM-CDs research from single herbal precursors to holistic compound formulas. This progress will facilitate the construction of diversified, characteristic ophthalmic nano-theranostic platforms and achieve deep integration between traditional ethnic medicine theories and nanotechnology.

## 6 Conclusion

TCM-CDs represent innovative functional therapeutic agents that integrate traditional Chinese medicine and nanotechnology. Their core innovative strength originates from intrinsic ocular therapeutic capacity, which does not rely on encapsulated exogenous drugs or residual herbal components. In animal models TCM-CDs are characterized by low toxicity and barrier-penetrating properties, multi-target therapeutic functions and nanozyme catalytic activity, showing potential for intervening in a wide range of refractory ocular disorders. However, this field still faces challenges including unresolved academic controversies, insufficient long-term safety data, lack of standardized systems and slow clinical transformation. Future research should advance simultaneously at fundamental and applied levels. On one hand, researchers need to further dissect functional mechanisms, optimize TCM-CDs nanostructures and establish unified quality control specifications. On the other hand, ethnomedicine- and compound formula-derived TCM-CDs should be prioritized as core research directions to develop innovative therapeutic platforms. On this basis, novel proprietary ophthalmic nano-drugs are expected to be developed, facilitating the coordinated development of precision ophthalmic therapy and the modernization of traditional medicine.
